# XXVII SESPO CONGRESS – Toledo - Spain: 30 September and 1 October 2022

**DOI:** 10.4317/jced.1122335667800

**Published:** 2023-11-01

XXVII SESPO CONGRESS – Toledo - Spain, 30 September and 1 October 2022, Abstracts of the scientific communications follow.

