# Supplementary material for: XXVII SESPO CONGRESS – Toledo - Spain: 30 September and 1 October 2022
Source: J Clin Exp Dent. 2023 Nov 1;15(Suppl 1):S1–S16. doi: 10.4317/jced.1122335667800 (PMC10624059; doi:10.4317/jced.1122335667800)
Supplement: Related texts

# Abstract: ORAL HEALTH INTERVENTION FOR RURAL SCHOOLS IN THE “SERRANÍA CONQUENSE” (2022)

**Authors:** M.G. Frontan Quevedo, F. García-Navas Romero, I. Bañón Anguix, R.M. Moya Díaz

**Affiliations:** *E-mail: gabyfrontan@yahoo.es; Primary care dentist at the Primary Care Health Centre of Priego. GAI Cuenca. Health Service of Castilla La Mancha (SESCAM), Spain.*

## Introduction

 Educational centres are an ideal environment to work on oral health. After two years of pandemic without being able to go out to the school community, we propose this activity to bring the PADI programme to all the schoolchildren in the Cuenca mountains attached to the Oral Health Unit (USBD) of Priego. 

## Objectives

 To motivate and make children in the Priego area responsible for their oral health. To provide teachers with materials to improve their training as oral health promoters. To mo- tivate families to come to the USBD. 

## Methodology

 The 13 rural cluster schools (CRAs) in the area were visited. An interactive workshop on health education and promotion of healthy habits was held in each of them. In order to evaluate the understanding of the messages conveyed, a drawing competition was proposed. Teachers were given a handbook to support them in carrying out further tasks in class on health education. Each child took home a recruitment letter, the telephone number and information on the USBD service portfolio. The activity ended with a meeting of all schools on 20 March in the auditorium of Priego to celebrate WORLD ORAL HEALTH DAY. 

## Results

 Children and teachers from all CRAs participated actively. The messag- es conveyed were clearly and creatively recorded in drawings. This intervention has increased demand in the USBD. 

## Conclusions

 A high level of participation was achieved. Motivation was reflected and increased patient recruitment to the USBD was achieved. More work needs to be done along these lines.

# Abstract: KNOWLEDGE ABOUT MOLAR INCISOR HYPOMINERALISATION (MIH) AMONG STUDENTS OF THE DEGREE IN DENTISTRY AND THE HIGHER TRAINING CYCLE IN ORAL HYGIENE IN THE VALENCIAN COMMUNITY

**Authors:** T. Almerich Torres, V. Tarazona Valero, J.E. Iranzo Cortés, J.M. Montiel Company, J.M. Almerich Silla

**Affiliations:** *E-mail: teresa.almerich@uv.es; Associate Professor of Preventive and Community Dentistry. University of Valencia, Spain.*

## Introduction

Molar Incisive Hypomineralisation (MIH) was first defined in 2001 as a dental tissue alteration whose pathognomonic signs are demarcated, asymmetrical lesions with qualitative enamel defects. As it is an emerging pathology, with an early onset and a worldwide prevalence of between 2.4 and 40%, it is necessary to analyse the theoretical and practical knowledge in the curricula of our universities and vocational training centres.

## Objectives

To assess the level of knowledge about the diagnosis and treatment of MIH in students of the Degree in Dentistry and Higher Vocational Training in Oral Hygiene (Training Cycle) by means of online questionnaires. 

## Methodology

Cross-sectional observational study using online questionnaires. A simple descriptive statistical analysis was carried out and an analytical statistical analysis by groups according to the group (Degree in Dentistry or Training Cycle), obtaining the Chi-square with its p-value together with the symmetrical Cramer’s V measure. 

## Results

 Of the 232 responses to the questionnaire, 210 were valid, of which 105 were from undergraduate students and 105 from vocational training students. The response rate was 51.8% and the effectiveness rate was 74.56%. 81.1% claimed to have heard of MIH although 54.8% confused it with other types of dental defects. 

## Conclusions

 The level of knowledge of MIH was higher in all the aspects evaluated among the students of the Degree in Dentistry than among the students of Oral Hygiene. There is a need to increase the theoretical and practical knowledge of MIH in the curricula of both degrees.

# Abstract: ORAL HEALTH DATA BANK SPAIN-UV, A NEW TOOL FOR VISUALISING EPIDEMIOLOGICAL DATA ON ORAL HEALTH IN SPAIN.

**Authors:** J.M. Montiel Company, J.M. Almerich Silla, A.W. Montiel Nicholas

**Affiliations:** *E-mail: jose.maria.montiel@uv.es; Department of Stomatology. University of Valencia, Spain.*

## Introduction

 Oral epidemiological data allow us to know the distribution of oral diseases in the population and to assess their evolutionary trends in order to determine treatment needs and the prioritisation of public resources. 

## Objectives

 For all these reasons, the aim was to develop a tool that allows rapid access to the main oral health indicators obtained from national epidemiological studies. 

## Methodology

An interactive web-app dashboard has been created, based on the R programming language with the help of the Shiny package, in which the official aggregate data published from oral health studies carried out in Spain have been introduced. 

## Results

The Data Dashboard, called ORAL HEALTH DATA BANK SPAIN-UV, allows the user to access the software via the Internet, which will function as a web page and will allow interactive visualisation of the indicators of caries, periodontal disease and other oral conditions of the five age cohorts explored. It will have interactive tables and graphs that can be adjusted according to variables such as cohort age, gender or socioeconomic status. In addition, it will allow epidemiological trends to be assessed, as well as the downloading of the graphs or tables generated on the web-app. 

## Conclusions

 ORAL HEALTH DATA BANK SPAIN-UV could be a useful tool to be used for teaching purposes by primary care dentists and for decision making in oral public health.

# Abstract: UNIVERSITY STUDENTS IN HEALTH SCIENCES AND THEIR ROLE IN HUMANITARIAN PROJECTS. KNOWLEDGE AND PERSPECTIVE OF FUTURE PROFESSIONALS IN THE WORLD OF VOLUNTEERING.

**Authors:** S. Giz Castelao, S. Abounaim Afourir, L.M. Junquera Gutiérrez, M.F. Escobedo Martínez, S. Junquera Olay, E. Barbeito Castro

**Affiliations:** *E-mail: gizcastelaosheila@gmail.com; University of Oviedo, Spain.*

## Introduction

 Non-governmental organisations (NGOs) for cooperation in dentistry aim to promote the improvement of oral health in the most disadvantaged regions. 

## Objectives

 To investigate the knowledge about global oral health and volunteering in dentistry of the students at the University Dental Clinic of Oviedo (CLUO). 

## Methodology

A survey was carried out among all the dental students at the University of Oviedo using the virtual platform “GOOGLE FORMS”. The survey was divided into 3 parts: 1) Demographic data, 2) Questions about knowledge of global oral health, and 3) Questions about knowledge of dental volunteering.

## Results

 Of the total number of students enrolled in the 2021-2022 academic year, 90 (93.7% of the total) completed the surveys in this study. None of the CLUO students had participated in volunteering, but up to 64.4% had considered volunteering. The level of knowledge oral health was inadequate. The most recognised dental NGO was “Dentists Without Borders” (65.5%). Significantly, students in the clinical courses were more aware of the NGO “Dentists Without Borders” and different strategies to improve oral health in the target populations. Conclusion: Respondents show a significant willingness to volunteer in dentistry. However, there are significant differences both in the motivations for volunteering and in the strategies for improving oral health proposed

# Abstract: INEQUALITIES IN ORAL HEALTH AMONG CHILDREN IN CATALONIA: AN ECOLOGICAL STUDY

**Authors:** M. Figueras Cabanes, J. Busquet Vila, A. Lopez Cano, C. Garriga Palau del Mas, R. Martí Lluch

**Affiliations:** *E-mail: mfigueras.girona.ics@gencat.cat; Can Gibert del Pla Primary Care Centre (GI-2), Catalan Institute of Health (ICS), Girona, Spain.*

## Introduction

 The relationship between morbidity, mortality and the social determinants in diﬀerent contexts is an established fact. Health inequalities during childhood have been studied in Catalonia in relation to 29 pathologies that were considered relevant. Tooth decay was not considered among those pathologies despite being highly prevalent. In Europe many studies have validated a relation between tooth decay and poverty, therefore we set out to research this relationship in Catalonia.

## Objectives

 Main goal: investigate the relationship between oral health variables and socioeconomic variables: percentage of immigration and Compound Socio Economic Indicator (CSI), an indicator regularly used by Primary Health Care (PHC) decision makers. Secondary goal: investigate the link between oral health prevention activities and socioeconomic variables. 

## Methodology

 Ecologic observational study of the child population from 6 to 14 years old assisted in 279 Primary Care Centres in Catalonia, which provide PHC assistance to 80% of the Catalan population. Regular ethic protocols have been followed (IDIAPJGol 21/259-P). In the study the correlation Pearson test is used to display the relation, direction and intensity between the diﬀerent variables. 

## Results

 All oral health variables except for the two with less dispersion in the values’ range showed a connection with socioeconomic variables; the more the social disabilities the worse the oral health. No relation could be established between the prevention activities and the socioeconomic variables. 

## Conclusions

The two socioeconomic variables studied can be considered an indirect indicator of oral health and could be used in PHC dentistry planning as well.

# Abstract: “STOP SUGAR STOP CARIES”, A HEALTH PROMOTION PROGRAMME SUPPORTED BY THE ALLIANCE FOR A CAVITY-FREE FUTURE

**Authors:** R. Tarragó Gil, L. Gil Barrue, D. Serrano Peris, M. Aza Pascual-Salcedo, M.J. Lallana Alvarez, E. Lain Miranda

**Affiliations:** *E-mail: rosatarrago.formacion@gmail.com; Dental hygienist at “Seminario” Primary Care Centre. Aragonese Health Service, Spain.*

## Introduction

 Dental caries is the most prevalent chronic noncommunicable disease and one of the main public health problems worldwide. Frequently drinking excessive amounts of sugarsweetened beverages is a risk factor for obesity, type 2 diabetes, cardiovascular disease and tooth decay. Health promotion and education activities are essential, and it is crucial to involve all professionals to bring about change. 

## Objectives

 Make the population aware of the relationship between sugar consumption and dental caries. Make known the presence of “hidden sugars”. Raise awareness among health personnel of the importance of promoting a healthy diet. 

## Results

 Three posters have been designed in infographic format. The main poster is an explanatory panel of hidden sugars in which snacks, processed foods and sugary drinks are displayed, which are an important source of empty calories. Next to each product is the equivalent number of sugar cubes.In addition, there is an image of how caries is produced, sugar recommendations according to the WHO, and some QRs that lead to another sign “stop caries watch”, and to a third panel with healthy food alternatives. Each poster has its explanatory video that can be viewed using a QR link. Another tool that has been designed and the population can download with QR is a guide to healthy micronutrients for teeth and the importance of food labelling to facilitate informed choices. In addition, the panels have been screened in eight continuing education courses, in three conferences and at a congress. All the posters, videos and Guide are posted on the websites of SESPO, SEOP, AHB Aragón, ACFF and Aragón School of Health. A dissemination campaign has been carried out on social networks of SESPO, SEOP, AHB Aragón, ACFF and the Aragonese health school. The panel is exhibited in all health centres in Aragon (123 centres). It has been disseminated in the media through three radio interviews, 1 press release and information on Aragón TV. In addition, 104 surveys have been carried out after viewing all the material. 98% of those surveyed relate the health risks of a diet rich in sugar. 87.5% have discovered new data on the real content of added sugar in foods. 75% consider themselves capable of interpreting food labels. 92% are willing to reduce foods with more than 10% sugar in their composition. 94% want to do without the consumption of sugary drinks. 86% know that consumption between hours increases the risk of caries. The level of satisfaction with the program has been 100%. Conclusion: There is great ignorance of the hidden added sugars in foods of habitual consumption. The programme presented in this work has eﬀectively made the population aware of the presence of hidden sugars and has promoted a change of habits in favour of health and prevention of caries.

# Abstract: ANTIBIOTIC PRESCRIBING IN DENTISTRY: A NATIONAL- WIDE CROSS-SECTIONAL STUDY.

**Authors:** M.A. Rodríguez Fernández, M. Zapata Cachafeiro, O. Vázquez Cancela, M. Mulero de Caso, E. Smyth Chamosa

**Affiliations:** *E-mail: almudena.rodriguez@usc.es; University of Santiago de Compostela, Spain.*

## Introduction

 Bacterial resistance is a public health problem in which many factors are involved and one of them is its indiscriminate use of antibiotics in the health sector. It is estimated that in Spain, dentists are responsible for 10% of antibiotic prescriptions at the community level.

## Objectives

 to analyse the antibiotic prescribing habits of dentists working in Spain and the resources they use to update their knowledge.

## Methodology

 a cross-sectional questionnairebased study was carried out addressed to registered dentists in Spain (N=38,072). The link was distributed through messaging services, social networks and websites of Scientiﬁc Societies and Professional Associations. 

## Results

 1,181 dentists participated and 73% (n=878) prescribe more than 5 antibiotics per month. Amoxicillin alone (61.2%) or combined with clavulanic acid (34.1%) and clindamycin (49.7%) in allergic patients are the antibiotics of choice. The main causes of antibiotic prescription are irreversible pulpitis (54.1%) or necrotic pulp with symptomatic apical periodontitis (52.7%) and incision and drainage of intraoral swelling (50.3%). The vademecum (70.6%) and the recommendations of Scientiﬁc Societies (33%) are the main sources of information on antibiotic prescription. 

## Conclusions

 Amoxicillin alone or combined with clavulanic acid and clindamycin for 4-7 days are the most prescribed antibiotics by dentists in Spain. The prescription of antibiotics does not always correspond to the indications in clinical guidelines. Vademecum and scientific articles are the main sources of information and updates on antibiotics.

# Abstract: COMMUNITY ACTIVITY TO PROMOTE ORAL HEALTH AND TEACH PRACTICAL ORAL HYGIENE IN SCHOOLCHILDREN AT HIGH RISK OF CARIES IN THE “DELTA DEL LLOBREGAT” PRIMARY CARE SERVICE

**Authors:** M. Terrades Oliver, E. Flores Gudiño, M.E. Giunta, S. Méndez Rodríguez, M.M. Sabater Recolons, M.T. Mateos Bueno

**Affiliations:** *E-mail: m.terrades.oliver@gmail.com; Primary Care Dentist at Primary Care Service (SAP) “Delta del Llobregat”. Catalan Institute of Health (ICS), El Prat de Llobregat, Barcelona, Spain.*

## Introduction

 The on-going high incidence of oral diseases, as well as their socioeconomic bias, requires an etiological approach focused on highrisk groups. Daily toothbrushing with fluoridated toothpaste is one of the most eﬀective measures to prevent caries and periodontal diseases. In Catalonia, the school program aimed at promoting tooth brushing does not include practical teaching of the brushing technique. 

## Objectives

 To design and implement a community activity to promote oral health and the practical teaching of tooth brushing techniques in schoolchildren at high risk of caries.

## Methodology

 1) A workshop is designed with a practical teaching of tooth brushing using plaque developer and toothbrushes for P5 children. 2) The two schools with the highest caries rate from each EAP are selected. 3) The satisfaction of the participating teachers is assessed through an anonymous survey. 

## Results

 6 of the 21 primary care dentists of the “SAP Delta del Llobregat” agreed to participate. The activity was carried out in 21 P5 classes from 9 schools (about 500 schoolchildren aged 5-6 years), without any incidence. The teachers’ satisfaction was 4.8 points on a scale from 0 to 5 (5 = maximum satisfaction). 

## Conclusions

 The activity shown was easily applicable. The satisfaction of the participating teachers was very high. More primary care dentists need to be involved in order to reach a larger number of schoolchildren.

# Abstract: URBAN-RURAL CONDITION IS A KEY FACTOR IN CARIES PREVALENCE AMONG SCHOOLCHILDREN IN MALLORCA

**Authors:** D. Vallejos Rojas, I. Coll Campayo, N. López Safont

**Affiliations:** *E-mail: d.vallejos@eua.edu.es; University School ADEMAUniversity of the Balearic Islands, Mallorca, Spain.*

## Introduction

 Health is conditioned both by individual local factors as well as by social, economic, environmental and geographic factors. Among the latter, the urban-rural condition plays an important role. Rural sectors have generally been associated with a lower socioeconomic level and a higher prevalence of diseases. 

## Objectives

 To study the prevalence of caries in schoolchildren in Mallorca according to the location of their educational centres, discriminated by rural (R) and urban (U) areas. 

## Methodology

 A cross-sectional observational epidemiological study was carried out in the school population of Mallorca, according to the manual Oral Health Surveys: Basic Methods (WHO 2013). Between Nov-2018 and Dec-2019, 717 schoolchildren from 1st, 6th and 4th ESO were explored in 28 educational centres, selected by stratiﬁed systematic random sampling. 

## Results

The DMFT index is higher in schoolchildren from rural areas (R: 0.87±0.11; U: 0.39±0.4 p<0.001) compared to schoolchildren from urban areas. In addition, these schoolchildren consume sugary soft drinks more frequently (R: 33.89 times/month ±1.28; U: 25.70 times/month ±1.19; p<0.001). Finally, we have observed that the educational level of parents/guardians is lower in rural areas (R: 6.08±0.13; U: 6.42±0.11 p=0.045; 1=no formal education, 2=less than primary, 3=ﬁnished primary, 4=ﬁnished secondary, 5=non-university studies, 6=university studies). 

## Conclusions

 In rural areas of Mallorca there is a higher prevalence of caries among schoolchildren compared to urban areas. As well as a greater consumption of sugary drinks. This may be related to a lower educational level of the parents/guardians.

# Abstract: DENTAL CARE POLICIES AND GENDER INEQUALITIES IN DENTAL VISITS AMONG OLDER PEOPLE IN CHILE (2006-2017)

**Authors:** M. Cornejo Ovalle, C. Garrido Urrutia, E. Raiqueo Vaccaro

**Affiliations:** *E-mail: mcornejo@odontologia.uchile.cl; DDS, MSc in Public Health. Faculty of Dentistry, University of Chile, Chile.*

## Introduction

 In 2006, the WHO called on countries to address the oral health of older people in ageing societies. Since then, Chile has implemented several public dental care policies and programmes aimed at bringing dental care closer to older people and reducing social inequalities in dental care. Two of these programmes are: 1) “More Smiles for Chile”, aimed at adult women; and 2) the implementation of oral health care for adults in their 60s with guaranteed access, timeliness, quality and financial protection (“Garantías Explícitas en Salud” or GES). 

## Objectives

to analyse the prevalence of visits to the dentist in people aged 60 and the inequalities according to gender, between 2006 and 2017, before and after the implementation of dental care policies and programmes aimed at older people in Chile.

## Methodology

 cross-sectional study through the use and secondary analysis of the databases of the National Socioeconomic Characterisation Surveys (CASEN) of Chile, which correspond to probabilistic samples of urban-rural national representation. The CASEN 2006 and 2017 surveys were used and because their respective measurements were carried out before and after the implementation of dental care policies and programmes aimed at older people; in addition, both surveys use the same methodology for measuring the variables studied. A sample of subjects aged 60 years was considered and dental visits and socioeconomic variables were used as explanatory variables. The association between categorical variables was verified through the Chi-square test (X2) and for the analysis of inequalities the prevalence ratio (PR) of dental visits was estimated according to the socioeconomic determinants studied. A 95% confidence level was considered with a p-value of 0.05. 

## Results

 the total prevalence of dental visits increased from 4.44% to 7.22% between 2006 and 2017 respectively. According to sex, prevalence increased statistically significantly from 3.87% (95%, CI: 2.87-5.1) to 5.77% (95%, CI: 4.58-7.16) in men, and from 4.93% (95%, CI: 3.85-6.2) to 9.37% (95%, CI: 7.97-10.91) in women, between the respective years analysed. The difference in prevalence between women and men observed in 2006 became statistically significant in 2017 (p<0.001). Gender inequalities became significant when assessing both crude and adjusted PR. For women, a crude PR of 1.6 (95% CI 1.24-2.11) and an adjusted PR of 1.67 (95% CI 1.28-2.19) were observed in 2017. In the adjusted model, the other variable that was statistically significant in 2017 was the most advantaged income quintile with an adjusted PR of 1.72 (95% CI 1.07- 2.75). 

## Conclusions

 the prevalence of dental visits increased significantly between 2006 and 2017 in the context of the implementation of dental care policies and programmes targeting older people. This increase was higher among women. A variation in gender-aligned inequalities was observed, although a social gradient also persists in other axes of the social inequalities analysed.

# Abstract: STUDY OF THE STATE OF ORAL HEALTH AND TREATMENT NEEDS IN PRIMARY HEALTH CARE Pérez Antona,IN THE PROVINCE OF ÁVILA

**Authors:** F.J. Pérez Antona, I.C. Pérez García, P. Pérez García, M. Sánchez Martínez

**Affiliations:** *E-mail: fjpadent@gmail.com; Primary care dentistry of the national health service of Castilla y León, Spain.*

## Introduction

 National Health Services should promote general health and oral health. 

## Objectives

To analyze oral health status, caries treatment needs and hygiene habits in a population aged 6 to 15 years in the province of Ávila and associated factors and to analyze the influence of immigration on oral health. 

## Methodology

 Observational, cross-sectional, descriptive study. An oral examination was carried out on 350 children aged 6 to 15 years, attended at the Oral Health Unit of the Health Centre of Las Navas del Marqués (Ávila). Sociodemographic variables (age, sex, social status, ancestry) and oral health variables were analyzed. 

## Results

 The dmft index in the 6-8 age group was 4.26. The percentage of children requiring treatment with one surface ﬁlling was 67.7%, with a mean number of surfaces to be ﬁlled of 2.06. The DMFT index in 12-15 age group was 2.69. A total of 56.8% of the children required one surface to be ﬁlled, with a mean number of surfaces to be ﬁlled of 2.34. Spanish children had a caries prevalence of 44.4% and immigrant children 77.1%. The CPI=0 in the 12-15 age group was 40.2%. Regarding brushing, Spanish children reported brushing more than once a day in 34.9%, while immigrants did it in 4.6%. 

## Conclusions

 Caries prevalence is higher than the levels reported in the Spanish national epidemiological study of 2020. Any population-based intervention should consider the social factors that enhance risk factors and reduce opportunities.

# Abstract: SOCIAL VULNERABILITY AND ORAL HEALTH IN THE “CAÑADA REAL GALIANA”. A CRITICAL ANALYSIS OF THE ORAL HEALTH OF THIS GROUP AS AN EXPRESSION OF INEQUALITY

**Authors:** j. Sánchez Ituarte, M. Lamas Oliveira, A. de la Hoz Calvo, M. Frías Senande, D. González Alarcón

**Affiliations:** *E-mail: julia.sanchez.ituarte@gmail.com; European University of Madrid, Spain.*

## Introduction

 Although in health sciences we talk about populations “at risk”, there has been a paradigm shift in sociological analysis, where risk is transcended, implementing the concept of vulnerability. 

## Objectives

 This study aimed to analyse the oral health vulnerability of a group of children aged 6-16 years. Thus, we described and compared the oral health status and access to health services of children aged 6-16 years in the Madrid deprived housing area of “La Cañada Real” and “El Gallinero”, comparing it to a similar aged population at a national and regional level.

## Methodology

 Cross-sectional observational study. WHO methodology for oral health studies (1997; 2013). Only the intra-examiner Kappa index >0.95 was considered consistent. Statistical analysis was carried out with IBM SPSS v.24. A total of 188/400 children were screened (50% of male and female). The study was approved by the Clinical Research Ethics Committee of the University Hospital Severo Ochoa (Madrid), with the informed consent from all parents/guardians of the children. 

## Results

 The percentage of children with dmft/DMFT index >0 is much higher in this population (89.7% at 5-7 years in primary dentition, 70.7% at 11-13 years in permanent dentition and 92.6% at 14-16 years). The presence of sealants in at least one permanent molar is strikingly low (3.7% at 14-16 years of age), being a treatment covered by the PADI programme of the Community of Madrid. Conclusion: Social vulnerability is an expression of inequality in levels of oral health and access to specialised care in this group, which presents significantly higher levels of pathology than other groups of similar age. We need a more social vision, capable of bringing health closer to this community and establishing specific protection tools for their type of vulnerability.

# Abstract: IMPACT OF THE IMPLEMENTATION OF AN OUTPATIENT CLINIC FOR PATIENTS WITH DISABILITIES IN JAÉN

**Authors:** A.J. Expósito Delgado, E. Calvo Pulido, E. Martos Cobo, G. Ortiz Cabrero, M.A. González Martínez

**Affiliations:** *E-mail: clinicaolavide@gmail.com; Dentist at the University Hospital of Jaén, Spain.*

## Introduction

 People with disabilities encounter barriers to ensure their oral health associated with different considerations ranging from the patient himself, to the dentist’s skills, social or political responsibilities. The rate of hospital referral for the care of these patients under general anaesthesia or deep sedation is very high. This care option is not free of risks and side effects for the patient, as well as being more complex and costly. As a result, access to these services is very restricted and the waiting list is very long. 

## Objectives

 To analyse the impact of the implementation of an outpatient clinic for the care of patients with disabilities.

## Methodology

 An observational and descriptive study was carried out on the healthcare activity of a new outpatient clinic at the University Hospital of Jaén for the care of patients with disabilities from November 2021 to June 2022. The variables analysed were age, sex, form of access, type of treatment, need for superficial sedation and local anaesthesia, caries risk, type of disability and number of referrals for general anaesthesia or deep sedation. 

## Results

 A total of 224 interventions were performed (66 in children <15 years and 158 patients ≥15 years). 39.7% of the procedures did not require any type of anaesthesia, 47.3% required local anaesthesia and 12.9% had to be referred for general anaesthesia or deep sedation. 29.9% of outpatient procedures required superficial sedation. Caries risk was high in 74.5% of the patients operated on. Patients with autistic spectrum disorder, Down’s syndrome and cerebral palsy were the most demanding. The surgical waiting list has been reduced by 97.23% in patients under 15 years and 86.61% in adults.

## Conclusions

 In our experience, outpatient care in a hospital environment for the disabled patient is comfortable, minimally restrictive, safe and efficient. It guarantees patient accessibility, favours the monitoring of their oral health and reduces patient referrals for surgery under general anaesthesia and deep sedation.

# Abstract: STUDY OF THE ORAL HEALTH TREATMENT PATHWAY ADOPTED BY THE PIGMEA BAKAL COMMUNITY IN SOUTH-EAST CAMEROON

**Authors:** D. González Alarcón, A. Mankongo, C. Pilipili, M. Ashu Agbor, B. Dongmo, C. Deube Ngako

**Affiliations:** *E-mail: david.gonzalez.alarcon@gmail.com; Dentist at Mindja Paul Health and Emancipation Research Centre. University of Yaoundé, Cameroon.*

## Introduction

 Access to oral health, understood from a Western medical conception, presents a scenario of inequity and inaccessibility for indigenous peoples in Cameroon. 

## Objectives

 The aim of this research was to learn about the oral health therapeutic itineraries used by the Baka population in the South of Cameroon following traditional and ancestral pathways. 

## Methodology

 Descriptive study carried out by the Health Department of the Association "Zerca y Lejo" and the Mindja Paul Health and Emancipation Research Centre. Data were collected during personal interviews in March and April 2021. Through semi-structured interviews with 51 traditional practitioners and 118 adults in 14 Baka Pygmy villages in the East and South region of Cameroon. 

## Results

 Access to traditional doctors represents the first option for 98.36 % of the population; only 19.6 % of traditional doctors are recognised by the government. Oral pathologies represent one of the eight most frequent pathologies treated by this medicine. Dental caries is the most treated process (66.6 %) followed by periodontal pathology (23.52 %), including to a lesser extent containment due to mandibular trauma and Cancrum Oris (5.8 %). 70.6 % of the traditional practitioners considered it positive to know the techniques of conventional dentistry. 

## Conclusions

 Access to health care for indigenous peoples and oral health in particular, requires a more complex approach committed to the indigenous concept of health. Knowledge of therapeutic itineraries is a functional tool of the first order for implementing an emancipatory community oral health plan. One of the possible perspectives is the implementation of a mixed health access programme, based on traditional health ﬁgures and promoting dentistry in the basic oral health package and implemented by community health leaders.

# Abstract: ACADEMIC STRESS AND HEALTH IN DENTAL UNDERGRADUATE STUDENTS

**Authors:** C.M. Arias Macías, J. Fernández Arias, J.F. Martin Morales, M. Bruna del Cojo, L. Prado Simón, I. Casado Gómez

**Affiliations:** *E-mail: cmargarita@ceu.es; CEU San Pablo University Madrid, Spain.*

## Objectives

 To identify the main triggers of stress and their relationship with general and oral health in students in the degree of dentistry at CEU San Pablo University. 

## Methodology

 A cross-sectional observational epidemiological study was performed, which included 114 students of the Degree in Dentistry at CEU San Pablo University. The Academic Stress Questionnaire at the University (CEU) developed by García Ros et al. was applied to determine the stress levels. In addition, self-perception of health was recorded using an ad hoc questionnaire. Both questionnaires were made available online using the virtual campus of the university. 

## Results

 The main causes of stress were the taking exams (64%), exposure to work and academic overload (50% respectively), and the lack of time to fulﬁl their academic obligations (46.4%). Students with a higher-grade point average show higher levels of stress in the presence of academic stressors, although without signiﬁcant diﬀerences (p=0.07). Regarding the self-perception of health, 87.7% of the students considered it good or very good although 32.5% felt that it has worsened after beginning their university studies. 40.3% of students had increased tobacco consumption, compared to only 27.2% of students who had increased alcohol consumption. Most of the students evaluated their oral health and oral self-care as good. 

## Conclusions

 The main stress triggers of students are the taking of exams as well as the lecture and academic overload. They evaluated their general health as good, although they felt it has worsened slightly since the beginning of their studies. The consumption of alcohol and tobacco has increased. They rated their oral health as good.

# Abstract: PERCEPTION OF THE TEACHING STAFF OF THE FACULTY OF DENTISTRY (UCM) ON THE USEFULNESS OF THE CAMBRA PROTOCOL

**Authors:** A.L. Lenguas Silva, O. Salgado Peralvo, J.F. Martín Morales, M.V. Mateos Moreno

**Affiliations:** *E-mail: alenguas@pdi.ucm.es; Faculty of Dentistry. Complutense University of Madrid, Spain.*

## Introduction

 The CAMBRA tool (Caries Management by Risk Assessment) is employed by students in the Faculty of Dentistry at the Complutense University of Madrid (UCM) as part of the Preventive and Community Dentistry (OPC) subject. Its purpose is to identify the risk of dental caries and implement individualized preventive protocols for patients seeking treatment. 

## Objectives

This study aimed to ascertain the opinion of the 5th-year teaching staff regarding the usefulness of CAMBRA in a project aimed at enhancing clinical care in OPC and involving the teaching staff in evaluation and quality control processes.

## Methodology

 The teachers of the OPC subject conducted an anonymous survey among the fifthyear teaching staff. Thus, eighteen questions were asked about the usefulness of CAMBRA, from which the four most relevant questions were selected. The responses were scored on a scale from 1 (strongly disagree) to 5 (strongly agree).

## Results

 Fourteen teachers participated in the survey. Considering the most positive scores (values 4 and 5), a very high percentage of respondents considered CAMBRA to be a useful tool to diagnose caries within the faculty (79%), to help students to develop patient review programmes (100%), to engage patients in self-care and motivate them to reduce their risk (93%) and to serve as a fundamental teaching tool in Cariology and Preventive Dentistry (79%). 

## Conclusions

 Overall, the results showed a generally positive opinion among faculty teachers who, regardless of their expertise, understand the importance of CAMBRA in preventive care and as an educational tool.

# Abstract: FROM COMPLEMENTARY FEEDING TO EATING AS A FAMILY, A PROPOSAL FOR INTERVENTION IN NUTRITIONAL EDUCATION

**Authors:** J. Busquet Vila, M. Figueras Cabanes, M. Busquet Figueras

**Affiliations:** *E-mail: jbusquet.girona.ics@gencat.cat; Montilivi/Vilaroja Primary Care Centre (GI-2), Catalan Institute of Health (ICS), Girona, Spain.*

## Introduction

 The health problem registration system in Primary Care has shown a prevalence of more than 15% of children who are either overweight or obese and a level of 50% of early childhood caries in the Vilaroja neighbourhood.

## Objectives

 To design an intervention aimed at empowering families in the development of a healthy and sustainable nutrition, considering both nutritional and emotional aspects for families with children up to the age of one year. 

## Methodology

Families (mothers, fathers, guardians) will be recruited through the Health Committee, paediatric teams, pharmacies and educational centres in the neighbourhood. Three participatory sessions of 40 minutes duration will be designed and key materials on different topics related to infant feeding will be provided. Additional reference materials will be sent via E-mail. 

## Results

 The contents of the prepared sessions are summarized. Session 1: calendar for the introduction of foods in the baby’s diet, cooking techniques and food presentation. Session 2: oral health, emphasizing the Hand Rule and Plate Rule. Session 3: key points from the guide "Small Changes for Better Eating", developed by the Department of Health of the Generalitat of Catalonia. Evaluation: The result of the interventions will be conducted using the Kidmed questionnaire to assess adherence to the Mediterranean diet; participants will also be invited to complete a satisfaction survey. 

## Conclusions

 The proposed community nutrition intervention program aims to reduce obesity levels in children and improve their health.

# Abstract: PARENT/GUARDIAN KNOWLEDGE OF ORAL HEALTH CARE FOR CHILDREN AGED 6-12 YEARS

**Authors:** V. Carballo Tejeda, V. Peinado Pelaez, I. Torremocha Macías, B. Carballo Tejeda, M.E. Lucerón Díaz-Ropero, A.M. Caleya Zambrano

**Affiliations:** *E-mail: vcarballotejeda@yahoo.es; Primary Care Health Centre “Isabel II”. Health Service of Castilla La Mancha (SESCAM), Spain.*

## Introduction

 Parental knowledge of oral health has a direct influence on the oral health of their children. Increased knowledge positively affects oral health and establishes the foundation for it to be maintained during adolescence and into adulthood. 

## Objectives

 To assess the knowledge of oral care among parents/guardians of children aged 6 to 12 years. 

## Methodology

 An observational, cross-sectional study was conducted. An anonymous questionnaire was sent to parents/ guardians of primary school children. The questionnaire consisted of 15 questions on oral care knowledge (5 questions on oral hygiene, 5 on diet and 5 on oral pathology). 

## Results

 A total of 310 responses were obtained, of which 85.6% were from women. Notably, 58.1% did not know the appropriate amount of fluoride toothpaste for children aged 6 to 12 years. Furthermore, 69.7% believed that toothbrushes should be replaced every 6 months and 63.5% believed that a child at primary school is capable of independently brushing their teeth. Additionally, 55.5% believed that a permanent tooth should be re-implanted in cases of avulsion. 

## Conclusions

 Based on the results obtained, we advocate for the implementation of educational sessions targeting parents to enhance their knowledge of oral care for their children. Furthermore, expanding the sample size would provide more representative insights into this subject matter.

# Abstract: SURVEY ON THE TREATMENT GUIDELINES FOR ORAL LICHEN PLANUS IN THE ORAL HEALTH UNITS OF THE SERMAS

**Authors:** M. Lamas Oliveira, M.V. Mateos Moreno, G. García Vicent, A.L. Lenguas Silva, A.I. Terán de Agustín, L. Piñas Caballero

**Affiliations:** *E-mail: martalamas@telefonica.net; Primary Care Health Centre “Pozuelo Estación”. Health Service of the Community of Madrid (SERMAS), Pozuelo de Alarcón, Comunidad de Madrid, Spain.*

## Introduction

 Oral lichen planus (OLP), a chronic inflammatory disease, is the most prevalent noninfectious oral pathology commonly treated by primary care dentists. However, there is a lack of clinical practice guidelines regarding its treatment and recommendations for the first-line medication with respect to dosage, frequency and the method of administration to be used. 

## Objectives

1) To assess the treatment approaches, dosage, frequency and perceived effectiveness of OLP treatments used by primary care dentists in Madrid. 2) To identify potential training and clinical consensus needs in managing OLP. 

## Methodology

An anonymous online survey using Google Forms was distributed to 197 primary care dentists in Madrid, investigating their practices in treating OLP. 

## Results

 Out of the 52 respondents, 90.4% of dentists exclusively used corticosteroids for symptomatic or erosive cases of OLP. Triamcinolone acetonide was the preferred medication used by 92.3%, albeit in widely varying concentrations. Around 69.2% applied it 2-3 times daily, with 71.2% formulating it in orabase and 26.9% in aqueous solution. Approximately 65% did not combine corticosteroids with other medications, while 19.2% utilized antifungal agents and the remaining dentists employed different drugs, particularly analgesics/anti-inflammatories and anaesthetics. As a second-line treatment, 65.4% used clobetasol propionate and 23.1% opted for fluocinolone acetonide. The perceived efficacy of corticosteroids, as rated by the professionals, was 6.7 on a scale of 1-10. 

## Conclusions

 1) Primary care dentists demonstrate agreement regarding the choice of medication for OLP, although there is variability in other treatment parameters. 2) Enhancing training on OLP and establishing clinical practice guidelines are imperative to ensure standardized care approaches.

# Abstract: PROFILE OF USERS OF COLOMA VIDAL SOLIDARITY DENTAL CLINIC

**Authors:** I. Coll Campayo, C. Lluy Gonzalez, P. Sánchez Tamayo, B. Juan Sánchez

**Affiliations:** *E-mail: irene@dentistassobreruedas.es; Coloma Vidal Solidarity Dental Clinic (Dentistas Sobre Ruedas), Palma de Mallorca, Balearic Islands, Spain.*

## Introduction

 Half of the population in the Balearic Islands struggles to make ends meet, while 22% are at risk of poverty. This has resulted in the Balearic Islands having the highest AROPE rate increase in the country (Spain). The majority of individuals receiving benefits to alleviate vulnerability are women. 

## Objectives

 To describe the profile of individuals attended at the Coloma Vidal Solidarity Dental Clinic (located in Palma de Mallorca, Balearic Islands, Spain) during the year 2021. 

## Methodology

 Cross-sectional observational epidemiological study conducted among users of the Dentistas Sobre Ruedas NGO project during 2021. A total of 1,075 individuals between the age of 4 and 92 years old were analysed in Mallorca.

## Results

 The clinic attended 633 females and 442 males, with an average age of 46.56 ± 1.07 years. Spanish nationals accounted for 66.23% of the sample, while 33.76% were foreign nationals. Approximately 49.04% were unemployed, 10.07% were retired and only 10.93% were employed. Among the unemployed, women had a higher average number of children compared to men (1.56 vs 1.31, p=<0.001). 

## Conclusions

 Oral health continues to be a significant concern that requires attention from government and public administrations. The female population appears to be disproportionately affected by poverty, highlighting the need for targeted interventions.

# Abstract: ANALYSIS OF FACTORS THAT REDUCE DENTAL ANXIETY

**Authors:** J.D. García Morales, Y. Mendoza Lemes, K. Frohn

**Affiliations:** *E-mail: javiergarmo94@gmail.com; Fernando Pessoa Canarias University (UFP-C), Las Palmas de Gran Canaria, Canary Islands, Spain.*

## Introduction

 Dental anxiety is a growing issue affecting 15% of the Spanish population. One approach to address this problem is through Environmental Psychology, which examines how behaviour influences the environment and, in turn, how the environment affects behaviour.

## Objectives

 This research aimed to analyze the environmental factors that alleviate dental anxiety in adult patients during dental appointments. Specifically, the study focused on investigating the impact of a restorative environment on dental anxiety. 

## Methodology

 The study included a sample of 60 patients from the university clinic of Fernando Pessoa Canarias University. Patients were assigned to either a restorative environment or a control box and completed two tests: the State-Trait Anxiety Inventory (STAI) and the Modified Dental Anxiety Scale (MDAS). 

## Results

 The results of the t-student test revealed significant differences between the two groups, indicating that patients in the restorative environment exhibited lower levels of state anxiety and dental anxiety compared to those in the control box. 

## Conclusions

The results of this study are in accordance with current literature: the design of healthcare spaces has a strong impact on people, their emotional state and their recovery; and this has also been demonstrated in the dental setting. Consequently, dentists can consider new variables that have beneficial effects on the adult patient’s state in relation to dental anxiety.

# Abstract: IMPRESSIONS ON THE BASIC ORAL HEALTH INFORMATION OF THE FAMILIES ATTENDING THE DENTAL CLINIC

**Authors:** I. Paradas Lara, D. Felipe Roncero, B. Payo Aranda, O. Lucas de Francisco, N. Martínez Jiménez, E. Martín Blázquez

**Affiliations:** *E-mail: i.paradas@hotmail.com; Primary Care Health Centre “Santa Isabel”. Health Service of the Community of Madrid (SERMAS), Spain.*

## Introduction

 Currently, it is known that the home environment is important in maintaining children’s oral health. To achieve this, families need to have access to proper healthcare support and information. The community dental care programme (Plan de Atención Dental Infantojuvenil) of the Health Service of the Community of Madrid (SERMAS) focuses on the prevention of oral diseases in children from 7 to 16 years of age. However, the experience of our dental unit reveals that several sectors of the population are still unaware of the services of this programme (and the first dental check-up is often late). 

## Objectives

 To assess the level of knowledge of families attending the Leganés Oral Health Unit about the services offered and their children’s basic oral hygiene care. 

## Methodology

An anonymous survey was carried out using multiple-choice questionnaires. A total of 100 families with children under 16 years of age and who were visiting our Oral Health Unit for the first time or who only visited irregularly (with an interval of more than one year between visits) were enrolled in this study. The data collected were analysed using SPSS software. 

## Results

 The results showed that only half of the respondents claimed to have been referred from paediatrics. In a similar proportion, respondents were unaware of the public dental service portfolio. In addition, there is also a lack of knowledge about the appropriate timing of the first dental checkup.

## Conclusions

 Although this survey needs to be strengthened by expanding the sample size and the questionnaire, the preliminary data obtained suggest a crucial need to improve communication between paediatrics (first point of contact with families) and public dental health services.

# Abstract: OROFACIAL SYMPTOMS AFTER ADMINISTRATION OF SARS-CoV-2 VACCINE: A SYSTEMATIC REVIEW

**Authors:** J.F. Martin Morales, I. Casado Gómez, A.L. Lenguas Silva, C.M. Arias Macías, E. Descalzo Casado

**Affiliations:** *E-mail: josefrma@ucm.es; Faculty of Dentistry. Complutense University of Madrid, Spain.*

## Introduction

 In late 2019, the SARS-CoV-2 syndrome emerged in Wuhan, China. The COVID-19 pandemic quickly spread worldwide, affecting millions of people. The development of safe and effective vaccines against SARS-CoV-2 has been rapid, but adverse effects have been reported following vaccination. 

## Objectives

 The objective of this study was to synthesize the available literature regarding orofacial repercussions after administration of COVID-19 vaccines in order to establish accurate diagnoses in patients. 

## Methodology

A literature search was conducted in PubMed and Cochrane Library using the keywords “COVID-19," "COVID-19 vaccines," and "orofacial manifestations." A total of 22 articles published between 2020 and 2022 were included. 

## Results and Conclusions

Orofacial manifestations following COVID-19 vaccination are rare and occur with low frequency. Facial signs described in the reviewed studies include changes in sensitivity, facial/ lip swelling and Bell’s palsy. Adverse oral reactions following vaccination include gingival and/or palatal inflammation, pain, stomatitis, aphthous ulcers, lingual depapillation and xerostomia. The symptoms are generally moderate and respond well to conventional treatments.

# Abstract: EFFECT OF TOOTHPASTES CONTAINING ACTIVATED CHARCOAL ON ENAMEL. SYSTEMATIC REVIEW

**Authors:** J.E. Iranzo Cortés, C. Sorzano Miras, T. Almerich Torres, J.M. Montiel Company, J.M. Almerich Silla

**Affiliations:** *E-mail: j.enrique.iranzo@uv.es; Department of Stomatology. Faculty of Medicine and Dentistry. University of Valencia, Spain.*

## Introduction

 One of the main concerns in dental aesthetics is the white colour of teeth, leading consumers to turn to commercially available whitening toothpastes. Activated charcoal toothpaste is currently a popular “at-home” whitening method, largely due to its presence on social media platforms. 

## Objectives

 This systematic review aimed to study the effect of activated charcoal used in the composition of toothpaste and dentifrice powders on enamel structure. 

## Methodology

A literature search was conducted in four databases, resulting in 160 articles. Out of these articles, eight were included in the qualitative synthesis. The quality of the studies was evaluated using the PEDro scale. The effects of activated charcoal toothpaste on enamel roughness (Ra), substrate loss, changes in enamel morphology, and enamel colour change were assessed.

## Results

 All studies agree that activated charcoal toothpaste has a negative effect on dental enamel, leading to enamel wear. Regarding its whitening effect, four of the selected articles observed a slight impact on tooth colour, which was clinically noticeable but not statistically quantifiable. 

## Conclusions

Activated charcoal has a harmful effect on enamel, causing greater substrate loss compared to conventional toothpaste. The observed colour change may result from the loss of dental structure rather than a true whitening effect.

# Abstract: SYSTEMATIC REVIEW OF PEDIATRIC MORBIDITY IN ORAL SURGERY AFTER GENERAL ANESTHESIA

**Authors:** A. López Velasco

**Affiliations:** *E-mail: lopez_anavel@gva.es; Clinical Department-Malvarrosa, Valencia Regional Ministry of Health, Spain.*

## Introduction

 Children with special health needs require appropriate techniques in behaviour management, conscious sedation (CS), or general anaesthesia (GA). In this patient profile, the use of GA is increasing. Promoting and educating oral health, early prevention in pregnant women and groups at-risk, especially disabled patients, is crucial. 

## Objectives

 To conduct a comprehensive review of the existing literature on postoperative dental morbidity after GA in dental care for paediatric patients with and without special health needs, including its frequency, characteristics and association with the intervention undertaken.

## Methodology

 Systematic review of articles published until 23/02/22 in PubMed, Web of Science, Cochrane library, and EBSCO, using the following strategy: (baby OR child OR adolescent) AND (oral surgical procedures OR dentistry, operative) AND anaesthesia, general AND postoperative complications.

## Results

 Common postoperative symptoms include pain, drowsiness, inability to eat, bleeding, depression, need for analgesics, cough, fever, nausea, vomiting, dizziness, sleep disturbances and weakness. The most frequent complication is pain (11 articles, 98.9%), inability to eat (85.5% in 3 articles), drowsiness (84%), bleeding (40% in 5 articles), and nausea (26% in 4 articles). 

## Conclusions

The use of GA for paediatric patients with special needs is increasing and although mild/ moderate complications are always present, there is limited follow-up of these patients.

# Abstract: ASSOCIATION BETWEEN PERIODONTITIS AND SYSTEMIC DISEASES. PROMOTING GENERAL HEALTH IN THE DENTAL OFFICE

**Authors:** G. Antúnez Mejías, C. Wansze Albarrán, A. Contreras Briones, F.J. Herrera Briones, M. Vivas Lois, S. Cruz Vázquez

**Affiliations:** *E-mail: cgc51210@gmail.com; Herrera Briones Dental Clinic. Málaga, Spain.*

## Introduction

 Periodontitis, a chronic inflammatory disease caused by bacterial infection, significantly affects the supportive tissues of teeth and ranks as the 6th most prevalent disease globally, impacting 11.2% of the world’s population according to the World Health Organization (WHO). Advanced periodontitis poses a public health challenge due to its association with non-communicable systemic diseases, resulting in increased healthcare costs. 

## Objectives

 The main objective of this study was to establish the relationship between periodontal disease and systemic inflammatory processes that contribute to increased morbidity. Additionally, the study aimed to develop a visual representation, in the form of a diagram, to educate and inform the population about disease prevention in dental offices. 

## Methodology

 A comprehensive narrative and comparative literature review were conducted by consulting the PubMed and Cochrane Library databases. The review included randomized controlled trials (RCTs), systematic reviews, meta-analyses, and consensus reports published within the last 10 years. 

## Results

 Ten articles were selected for analysis. The findings indicate a significant association between poor periodontal health and the development of various diseases, attributed to the dissemination of periodontal pathogens causing chronic inflammation and elevated C-reactive protein levels. Periodontitis was found to influence and exacerbate conditions such as diabetes mellitus, cardiovascular disease, Alzheimer’s disease, and adverse pregnancy outcomes. Pregnant women with periodontal disease were at an increased risk of experiencing pre-eclampsia, preterm birth, and low birth weight. Although periodontal treatment during pregnancy was deemed safe and improved maternal oral health, the evidence supporting a decrease in the risk of adverse outcomes remained insufficient. Diabetes mellitus and periodontal disease exhibited a bidirectional relationship, serving as risk factors for each other’s onset and progression. Moreover, infectious gum pathology was found to heighten the risk of developing diabetes and compromise glycemic control in individuals with preexisting diabetes. Notably, periodontal treatment was shown to enhance glycemic control in patients with both periodontitis and diabetes. Individuals with periodontal disease exhibited a 15% increased risk of cardiovascular disease, as inflammation promoted the development and progression of atheromatous plaques. Additionally, periodontitis was associated with reduced endothelial function, which could be improved following periodontal treatment. The bacteria associated with periodontitis secreted pro-inflammatory cytokines and endotoxins that, upon entering the bloodstream, induced chronic brain inflammation and subsequent neuronal death. Consequently, periodontal disease emerged as a significant risk factor for neurodegenerative diseases, such as Alzheimer’s disease. Periodontal treatment held promise in slowing down the progression of Alzheimer’s disease. 

## Conclusions

 Dental offices play a crucial role in actively preventing non-communicable systemic diseases by prioritizing the treatment and control of periodontal disease, addressing shared risk factors, and providing guidance for a healthy lifestyle. Raising awareness and understanding regarding the intricate relationship between periodontal health and systemic diseases is vital for promoting overall well-being among individuals globally.

# Abstract: SERIAL TOOTH EXTRACTION IN PRIMARY CARE: A SOLUTION TO OVERCROWDING IN LOW-INCOME POPULATIONS

**Authors:** J. Manso Mora

**Affiliations:** *E-mail: xmm75@yahoo.es; Reus V Primary Care Centre. Catalan Institute of Health (ICS), Reus, Tarragona, Spain.*

## Introduction

 Serial extraction is a therapeutic approach employed in orthodontics to address significant disparities between bone and dental size, leading to dental crowding and malocclusion. While typically performed as a preliminary step to orthodontic treatment, it can also be a valuable option for patients with limited financial resources to afford private treatment. This communication presents a described protocol for serial extraction, along with two clinical cases. 

## Objectives

The objective of this study is to evaluate the effectiveness of serial extraction as an interceptive treatment for large bone-dental discrepancies, resulting in proper occlusion. Additionally, the study aims to explore the potential benefits of serial extraction in improving dental alignment and occlusion in patients who cannot afford orthodontic treatment due to financial constraints. 

## Methodology

The study includes a descriptive analysis of the serial extraction protocol and presents two clinical cases to illustrate its application. The protocol and clinical outcomes were evaluated based on the established criteria for dental alignment, occlusion, oral hygiene, and patient satisfaction.

## Results

 Serial extraction demonstrated efficacy in treating large bone-dental discrepancies and achieving favourable occlusion. Furthermore, it showed potential to significantly improve dental alignment and occlusion in patients with limited financial resources for orthodontic treatment. Improved dental alignment facilitated better oral hygiene, reduced the likelihood of malocclusion impacting mastication or temporomandibular joint health, and provided psychological benefits by enhancing aesthetics. 

## Conclusions

 Serial extraction serves as an effective interceptive treatment for addressing large bone-dental discrepancies and achieving proper occlusion. It can be a valuable option for patients unable to afford orthodontic treatment, offering significant improvements in dental alignment and occlusion. As X-rays and dental extractions are covered by the Spanish public health service, serial extraction can be performed in primary care centres. However, it is essential to provide training to dentists in the public health service to identify and treat patients who may benefit from serial extraction as a costeffective approach to improving dental alignment and occlusion.

# Abstract: SOLITARY BONE PLASMACYTOMA AT THE MANDIBULAR LEVEL: ABOUT A CLINICAL CASE

**Authors:** E. Flores Gudiño, H. Viñals Iglesias, A. Lozano Porras, N. Flores Gudiño, M. Sabater Recolons

**Affiliations:** *E-mail: evafloresgudi@hotmail.com; Disset de Setembre Primary Care Centre. Delta Oral Health Unit. Catalan Institute of Health (ICS), El Prat de Llobregat, Barcelona, Spain.*

## Introduction

 Plasma cell neoplasms encompass a range of diseases characterized by the excessive production of abnormal plasma cells or myeloma cells, leading to the formation of tumors in bones or soft tissues. One subtype is plasmacytoma, where abnormal plasma cells accumulate in a single location and form a tumor. Solitary bone plasmacytoma, although rare in the head and neck region (0.4%), predominantly affects long bones and the axial skeleton. 

## Case Report

 A 62-year-old male patient presented to the Primary Care dentistry service with complaints of poor occlusion with his complete dentures, which he had been wearing for 2 years. Clinical examination, including intraoral and extraoral assessment with a focus on temporomandibular joint dynamics, revealed significant bone expansion in the fourth quadrant. The patient experienced masticatory difficulties and aesthetic concerns but reported no associated abnormalities in other body locations. Orthopantomography revealed a large radiolucent lesion affecting the right hemimandible. The case was referred to the Head and Neck Functional Unit for further evaluation, with ongoing follow-up provided by the primary care dentistry service. 

## Conclusions

 Primary care dentists play a crucial role in the early diagnosis of oral cavity pathologies, even those that are rare. Their vigilance can potentially save lives and enhance the quality of life for patients.

# Abstract: INTERCEPTIVE ORTHODONTICS IN PRIMARY CARE DENTISTRY

**Authors:** M.E. Rodríguez Priego, M. Moreno Martínez, C. Costela Serrano

**Affiliations:** *E-mail: estherrodriguezpriego@hotmail.com; Primary Health Centre of Motril Centro, Granada South Health Management Area - Junta de Andalucía. Motril, Granada, Spain.*

## Introduction

 The primary care odontostomatology services encompass a range of general provisions that are not always effectively implemented. This situation prompts dentists to consider the importance of carrying out caries prevention and treatment, as well as providing basic diagnostics, family education, and, when feasible, addressing malocclusions in primary and mixed dentition that can impact a child’s chewing ability and dental arch development. 

## Case Report

 An 8-year-old patient was treated at the Critical Care and Emergency Unit, Paediatrics, and Primary Care Dentistry for dental pain. The patient had cognitive deficits due to congenital hypothyroidism (EUTIROX) and dysmorphic features, with a negative Array test and a 33% disability. Both parents had intellectual disabilities at a 67% level. The patient exhibited severe caries lesions and fistulas in teeth 51-52, along with a crossbite in teeth 11-12. The proposed treatment plan involved the extraction of teeth 51-52 and sealants for teeth 16-26-36-46. Oral hygiene education and hygienic-dietary habit changes were provided. Active follow-up with quarterly reviews was established, along with an indication for conservative treatment of primary teeth and urgent orthodontic intervention. Due to limited economic resources, interceptive orthodontics was suggested as a means to uncross teeth 11-12 using direct composite tracks in two phases. The procedure, risks, and benefits were explained, and the patient’s family agreed to proceed with the treatment. 

## Conclusions

 Following the first phase of treatment, which successfully uncrossed tooth 11, improvements in occlusion, arch perimeter, and a reduction in upper dental bone discrepancy were observed. It is crucial to implement simple interceptive orthodontic treatments to guide dental growth in the jaws, thereby preventing unilateral chewing and mandibular deviations.

# Abstract: ORAL MANIFESTATIONS IN CROHN’S DISEASE: A CASE REPORT

**Authors:** M. Aguilar Roldán, A. López García, O. García López, M.E. Lucerón Diaz-Ropero

**Affiliations:** *E-mail: martaaroldan@yahoo.es; Sonseca Health Centre, Toledo. Health Service of Castilla La Mancha (SESCAM), Spain.*

## Introduction

 Crohn’s disease is a chronic inflammatory condition of the digestive tract that can manifest in various extraintestinal areas, including the eyes, joints, mucocutaneous tissues, and bile ducts. Oral manifestations of Crohn’s disease can include aphthous-like ulcers, lip edema, cobblestone mucosa, and mucous tags. 

## Case Report

 A 39-year-old patient diagnosed with inflammatory-stenosing Crohn’s disease in 2016, with associated spondyloarthropathy. In 2021, the patient experienced a symptomatic colitis flare-up triggered by work-related anxiety. In May 2022, the patient sought consultation with their primary care physician due to significant joint involvement in the knees and wrists, a lip injury, oral mucosal aphthous ulcers, and target-like lesions on the hands. After evaluation of the herpetiform aphthous-like lesions by the dental clinic, the primary care physician consulted dermatology, which diagnosed the patient with probably herpes oral repetitive infection-triggered multiform exudative erythema. Treatment was initiated with Valacyclovir 500mg and Aloclair gel. 

## Conclusions

Remission of oral symptoms was achieved after one week of treatment with Valacyclovir 500mg. The collaboration between primary care and specialized care units facilitated a faster and more effective diagnosis, management, and treatment, ultimately benefiting the patient.
